# A novel point mutation within the EDA gene causes an exon dropping in mature RNA in Holstein Friesian cattle breed affected by X-linked anhidrotic ectodermal dysplasia

**DOI:** 10.1186/1746-6148-7-35

**Published:** 2011-07-08

**Authors:** Maria Gargani, Alessio Valentini, Lorraine Pariset

**Affiliations:** 1 Department for Innovation in Biological, Agro-Food and Forest systems (DIBAF), University of Tuscia, Viterbo, Italy

## Abstract

**Background:**

X-linked anhidrotic ectodermal dysplasia is a disorder characterized by abnormal development of tissues and organs of ectodermal origin caused by mutations in the *EDA *gene. The bovine *EDA *gene encodes the ectodysplasin A, a membrane protein expressed in keratinocytes, hair follicles and sweat glands, which is involved in the interactions between cell and cell and/or cell and matrix. Four mutations causing ectodermal dysplasia in cattle have been described so far.

**Results:**

We identified a new single nucleotide polymorphism (SNP) at the 9^th ^base of exon 8 in the *EDA *gene in two calves of Holstein Friesian cattle breed affected by ectodermal dysplasia. This SNP is located in the exonic splicing enhancer (ESEs) recognized by SRp40 protein. As a consequence, the spliceosome machinery is no longer able to recognize the sequence as exonic and causes exon skipping. The mutation determines the deletion of the entire exon (131 bp) in the RNA processing, causing a severe alteration of the protein structure and thus the disease.

**Conclusion:**

We identified a mutation, never described before, that changes the regulation of alternative splicing in the *EDA *gene and causes ectodermal dysplasia in cattle. The analysis of the SNP allows the identification of carriers that can transmit the disease to the offspring. This mutation can thus be exploited for a rational and efficient selection of unequivocally healthy cows for breeding.

## Background

Ectodermal dysplasia (ED) is a genetic disease characterized by abnormal development of tissues and organs of ectodermal origin, including teeth, hair, nails and sweat glands [1]. There are different forms of ED, the most common of which is caused by mutations in X-linked ectodysplasin gene A (*EDA*). It has been described in human [2], mouse [3], dog [4] and cattle [5]. Common features shown by the affected individuals are hypodontia, sparse hair and absence of sweat glands.

Numerous mutations in the X-linked *EDA *gene have been identified as the cause of disease in human [6-8] and mouse [3,9].

In cattle, the gene *EDA *is located on chromosome X q22 [10] and encodes ectodyslpasin-A, a membrane protein expressed in keratinocytes, hair follicles and sweat glands, which is involved in the interaction between cell and cell and/or cell and matrix. The *EDA *gene may encode for two protein isoforms, EDA1 and EDA2, that differ for the presence or absence of two aminoacids [5]. These two isoforms are members of TFN family.

Since the disease follows X-linked recessive transmission, only males present the full form, while heterozygous females (carriers) are asymptomatic or show slight symptoms, such as hypotrichosis and reduction in the number of teeth. The genetic transmission of this disease in cattle was described by Drögemüller and collaborators [5], who found a deletion of the whole exon 3 of *EDA *gene in an affected German Holstein calves and proposed to use for the bovine disease the name of the homologous human syndrome. The same authors subsequently described a G/T mutation at the beginning of intron 8 that leads to a defect in splicing and an in-frame protein deletion of 51 or 45 base pairs with respect to the EDA1 and EDA2 transcripts [11] and a C/T SNP at position 24 of exon 6 that causes a nonsense mutation of arginine (R) into a stop codon (X) [12].

Recently a new case of ectodermal dysplasia was reported in Danish Red Holstein cattle by Karlskov-Mortensen and collaborators [13]. They found a new transcript variant including an insertion of 161 bp LINE fragment between exon1 and exon 2.

In our study we screened the *EDA *gene in two affected calves and some of their close relatives, for a total of eight animals, in order to identify the mutations causing the disease in Holstein Friesian cattle breed.

## Results

**Figure 1:**
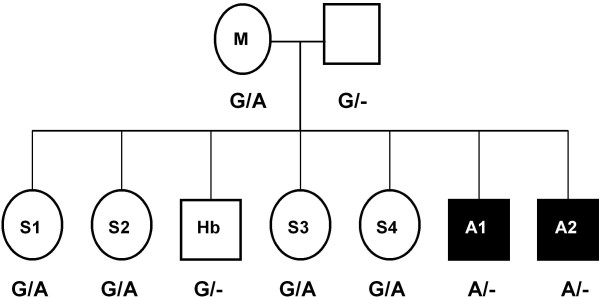
**Pedigree of the Friesian cattle family used in this study**. Pedigree includes the genotypes at the mutation site of *EDA *gene. Animals affected with anhidrotic ectodermal dysplasia are shown as solid symbols. Affected calves (A1, A2) were hemizygous A/-, the mother (M) and four sisters (S1, S2, S3, S4) were heterozygous G/A, the healthy brother (Hb) was hemizygous G/-. The pedigree is consistent with an X-chromosomal, recessive trait.

**Figure 2:**
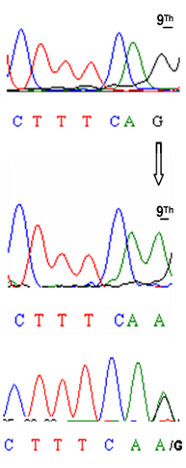
**Sequence analysis of exon 8 from wild, carriers and affected animal**. The upper sequence is from a healthy animal, the intermediate sequence is from affected calf and the lower sequence is from an affected calf. Arrow indicates the G/A mutation located at position 9 of bovine *EDA *exon 8.

Cattle samples were screened for mutations through DNA and RNA sequence analysis. By sequencing the DNA of the sampled individuals, we identified a single nucleotide polymorphism (SNP) G/A at the 9^th ^base of exon 8 [GenBank: AJ278907.1 position 30.549]. Both affected calves were hemizygous A/-, the mother and the four sisters were heterozygous G/A (therefore all carriers), the healthy brother was hemizygous G/- (Figure 1,2). To verify if the mutation had effects on exon splicing we performed an analysis using Human Splicing Finder software [14]. The results revealed that the mutation is located within the exonic splicing enhancer (ESE) recognized by SR proteins.

**Figure 3:**
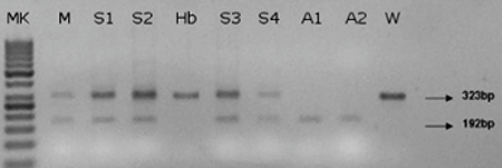
**Agarose gel electrophoreses of RT-PCR products from exon 6 to exon 9 in a Friesian cattle family**. The wild type (W) and the healthy brother (Hb) showed a single band of 323 bp. The affected calves (A1, A2) showed a band of 192 bp. The mother (M) and the four sisters (S1, S2, S3, S4), all heterozygous, presented both bands. (MK) 50 bp ladder, arrows show the two bands of 192 bp and 323 bp.

**Figure 4:**
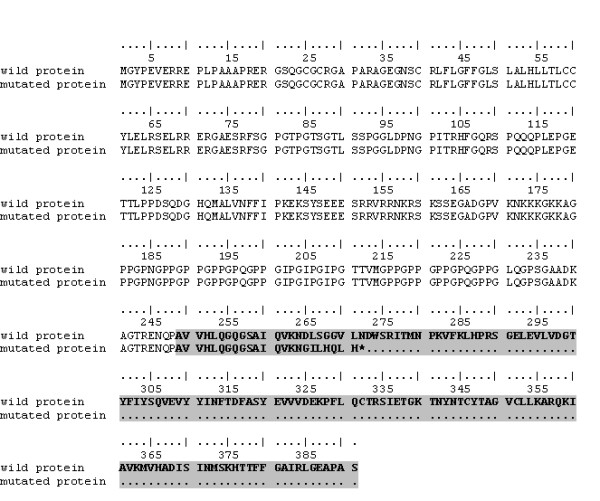
**Alignment of EDA amino acid sequence in affected and healthy cattle**. The lack of exon 8 in affected animals leads to a frameshift and a premature stop codon during translation process. The TNF domain is shown in grey.

At the RNA level we performed reverse transcriptase PCR (RT-PCR) and sequencing of the cDNA. In the affected calves a deletion in the amplified band of 323 bp, shown by healthy animals (Figure 3), compatible in size with the skipping of the whole exon 8 (131 bp), was observed. The mother and the four sisters presented two bands of 323 bp and 192 bp, corresponding to those of healthy and affected individuals respectively. Sequencing of the RT-PCR products revealed that the amplified fragment of the affected animals lacked exactly 131 bp, corresponding to exon 8. At the protein level, the exon skipping leads to a frameshift and consequently to a premature stop codon. The mutated protein is lacking in 119 amino acids (about half of the protein) including the TFN domain (Figure 4).

## Discussion

The identification of mutations causing a genetic disease is of great economic importance as it allows diagnosing the healthy carriers, and then the eradication of the disease. In this study we identified a new mutation in the bovine *EDA *gene causing ectodermal dysplasia in Holstein Friesian cattle breed.

The G/A transversion we found in the affected calves causes a change from the glycine residue encoded by the wild type allele to a serine residue. This missense mutation changes both amino acid sequence and protein structure causing deleterious effects. Several studies have shown that some missense mutations, located in exonic splicing regulatory elements, can cause diseases by altering the splicing machinery process [15]. The mutation we identified causes the incorrect splicing of both gene isoforms (EDA1, EDA2). In the mutated individuals the whole exon 8 is excluded from the final mRNA sequence during the splicing process.

The accurate removal of introns from pre-mRNA is essential for correct gene expression. Splicing is primarily regulated by splice-site motifs between intron and exon junctions [16]. In addition to the splice-site motifs, several studies have shown that other sequences are involved in the regulation of splicing process [17-19]. They form two classes of regulatory elements: exonic splicing enhancers (ESEs), recognized by SR proteins, and exonic splicing silencers (ESSs), recognized by hnRNP proteins [18-21]. These sequences have been identified by experimental and computational approaches [22].

In our study, the analysis of splicing revealed that the mutation is located within the exonic splicing enhancer (ESEs) sequences recognized by SRp40 protein. The function of SR proteins is binding to exonic sequences and enhancing the identification of the flanking splice site [23].

Several studies have demonstrated that single point mutations in exonic splicing enhancers (ESEs) lead to disease development [24-26]. For example, a C/T mutation in ESE sequence found in the human mitochondrial acetoacetyl-CoA thiolase gene results in exon 10 skipping. The protein is no more functional causing the mitochondrial acetoacetyl-CoA thiolase (T2) deficiency disorder [24]. Two silent substitutions in the Pyruvate dehydrogenase complex *(PDHA1 *gene) found in most patients with PDHc deficiency cause exon 5 skipping by disruption of a putative exonic splicing enhancer [25].

**Figure 5:**
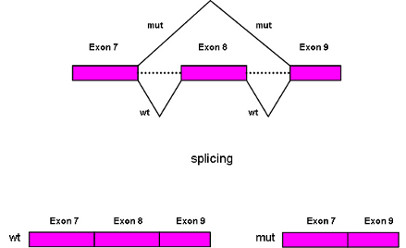
**A gene model**. Splicing patterns in wildtype (wt) and affected (mut) animals. In the wild animals the splicing process leads to a normal transcript. In the affected animals the G/A mutation influences the splicing process producing a truncated transcript in which exon 8 is skipped.

The EDA protein consists of a TFN domain for binding to the receptor, a collagen domain and cutting sites for furin. The TNF domain sequence is strongly conserved between cattle, human, mouse and dog demonstrating the presence of a very important motif in this region. The mutation we discovered influences the splicing process leading to the formation of a truncated protein. In the individuals presenting the described G > A transversion, SRp40 protein is no longer able to recognise the ESE sequence resulting in skipping of exon 8 (Figure 5). At the protein level, this leads to a frameshift deletion causing the loss of the TFN domain in the mutated ectodysplasin. The truncated EDA protein is no longer functional thus causing the ectodermal dysplasia disease.

Recently a mutation that leads to a truncated EDA protein was reported in Danish Red Holstein cattle. This mutation is an insertion of a LINE element between exon1 and exon2 in the EDA transcript, causing a frameshift which introduces a premature stop codon in the beginning of exon 2 [13]. Our results are in agreement with the data of Drögemüller *et al*. [11], reporting a point mutation within the 5' splice site of intron 8 in cattle *EDA *gene. The mutated transcript described by those authors uses a cryptic internal splice acceptor site within exon 8. The mutation we identified is located within the same cryptic splice site: this is a further confirmation supporting the involvement of the SNP we described in the splicing process.

## Conclusion

We identified a new single nucleotide mutation that causes ectodermal dysplasia in Holstein Friesian cattle breed. The analysis of this SNP allows the identification of the three possible genotypes (healthy, affected and carrier) and thus can be used to highlight carrier cows that can transmit the disease to the offspring. Ectodysplasia causes a significant economic loss: the use of heterozygous carriers in breeding results in a 25% chance of birth of an affected animal and 25% of birth of a carrier. Therefore, the early screening for heterozygotes that are carriers of the described mutation but do not exhibit the disease would particularly useful.

## Methods

### Samples collection

**Figure 6:**
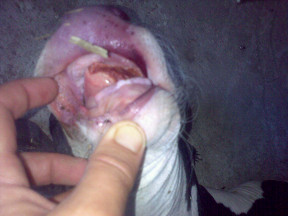
**Affected calf**. Complete hypodontia and generalized hypotrichosis in affected calf.

**Figure 7:**
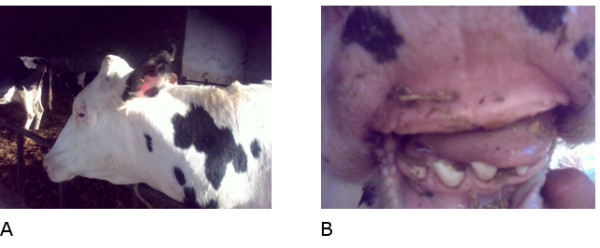
**Mother**. (A) Deficiency of the hair coat around ears and (B) partial hypodontia in the heterozygous mother.

Eight animals (two affected males, their mother, a healthy brother and four sisters), plus one healthy control of Friesian breed, were screened for mutations within the *EDA *gene. The affected animals showed complete hypodontia and a generalized hypotrichosis with a short hair coat (Figure 6). The severity of hypotrichosis varied in different areas of the body: both animals showed a short hair coat on the head, around eyes and on the tail. Moreover the neck, the flanks and the ventral abdomen appeared completely hairless. The skin was wrinkled especially on the neck. The absence of hair coat caused chronic skin problems such abrasion in both animals. These excoriations were more severe on the back. The mother showed mild symptoms of disease. We observed a partial hypotrichosis and hypodontia. The deficiency of the hair coat was more severe around ears while hair coat was normal on the flanks. Concerning dentition, we found only four incisors instead of eight and all teeth were long and pointed (Figure 7). Molars were normal in number and morphology. Moreover, one female showed an abnormal dentition with a partial hypodontia. We observed only four incisors bad positioned while the hair coat was normal. All other females are phenotypically sane.

Blood samples were collected in EDTA tubes and frozen at -20°C until extraction. Genomic DNA was isolated using DNeasy Blood & Tissue Kit (Qiagen), checked for DNA quality on agarose gel and quantified using a DTX microplate reader (Beckman Coulter) after staining with Picogreen (Invitrogen).

Skin samples were put in RNAlater (Sigma) immediately after collection and stored at -80°C. Total RNA was extracted from skin and blood using RNeasy tissue Kit (Qiagen) and RNeasy blood Kit (Qiagen) respectively and quantified using a DTX microplate reader (Beckman Coulter) using Quant-it RNA assay (Invitrogen).

Blood and skin samples collection were performed according to the Animal Ethics Committee of CRA, Italy, in agreement with local ethical requirements.

### Mutation analysis

Using a forward (5' TGGGGGTTGTGTACAG 3') and a reverse (5' TCAGCCATTGGCTGGTCTGGGC 3') primer located in intron 7 and intron 8 respectively [5], each PCR reaction was performed in 25 ul containing 10 ng of genomic DNA, 0.2 mM dNTP, 20 pmol of each primer, 1X buffer and 2U *Taq *polymerase (Bioline). After a 5 min initial denaturation at 94°C, 30 cycles of 30 sec at 94°C, 1 min at 55°C and 1 min at 72°C were carried out. After exosap (USB) purification, the PCR products were sequenced from both directions using the same primers with a CEQ8800 sequencer (Beckman Coulter) using DTCS kit (Beckman Coulter) according to manufacturer's instructions.

RT-PCR one step (Qiagen) was performed on total RNA using a forward primer (5' ATAAAGCTGGAACTCGAG 3') located in exon 6 and a reverse primer (5' TTGCCTGTCTCAATACTG 3') located in exon 9 [11]. The reaction was performed in 25 ul containing 10 ug RNA, 1X Buffer, 0.2 mM dNTP, 0.6 uM of each primer and 1 ul RT-PCR Enzyme (Qiagen). After a 30 min reverse transcription at 50°C and 15 min at 95°C, 30 cycles of 30 sec at 94°C, 1 min at 55°C and 1 min at 72°C were performed.

The RT-PCR products were sequenced from both directions using the same primers as described above.

Sequences analysis and alignment were performed using Bioedit software [27].

### Splicing analysis

In order to study the effects of mutations on splicing signals we used Human Splicing Finder Version 2.4.1 [14] available at http://www.umd.be/HSF/. The software provides a tool to predict the effects of mutations on splicing signals and to identify splicing motifs in the sequence of interest.

## Authors' contributions

MG carried out the molecular genetic studies and drafted the manuscript. AV participated in design and coordination of the study. LP carried out the design of the study and helped to draft the manuscript. All authors read and approved the final manuscript.
